# A commentary on ‘Short-term outcomes following intracorporeal vs. extracorporeal anastomosis after laparoscopic right and left-sided colectomy: a propensity score-matched study’

**DOI:** 10.1097/JS9.0000000000001217

**Published:** 2024-02-16

**Authors:** Tingfen Han, Shuai Liu

**Affiliations:** a Department of Traditional Chinese Medicine; b Department of Gastrointestinal Surgery, Taizhou Hospital of Zhejiang Province, affiliated to Wenzhou Medical University, Taizhou, People’s Republic of China

*Dear Editor*,

During laparoscopic colectomy, intracorporeal anastomosis (IA) is divided into left and right colon. Left-sided colonic anastomosis faces more complexities, but clinical research data is limited^1^. Teramura *et al*. designed a propensity score-matched study that reviewed 226 eligible patients undergoing elective laparoscopic colectomy. The study emphasized IA on both sides of the colon to assess the therapeutic difference between it and extracorporeal anastomosis (EA)^2^. It is concluded that IA during laparoscopic colectomy, especially in the left colon, can reduce the potential risk of postoperative complications.

‘Minimally invasive’ and even ‘non-invasive’ are the trends of modern surgery. Natural orifice specimen extraction surgery (NOSES) has attracted attention due to its enormous minimally invasive effect, especially in gastrointestinal surgery^3^. However, IA during laparoscopic colectomy is the prerequisite for NOSES^4^. This conclusion provides supportive evidence for promoting NOSES and will continue to promote the development of enhanced recovery after surgery (ERAS). We highly appreciate this clinical study but have questions about some details.

Firstly, further subgrouping on different surgical strategies is recommended for patients undergoing left-sided colectomy. Due to factors such as residual bowel length, anastomotic tension, and blood supply, left-sided colonic anastomosis faces more complexities, which affects the early healing of patients^1^. The authors concluded that IA is more favorable in some patients who have difficulty with anastomosis due to insufficient mobilization but did not clearly state the management of such anastomotic difficulties. After partial mobilization, performing anastomosis with almost no tension may be possible. For example, the residual colon is anastomosed without releasing the bowel and mesentery. Another possibility is to release sufficient mobilization before performing tension-free anastomosis. For example, when direct anastomosis is not possible after laparoscopic descending colectomy, further release of the mesentery and splenic flexure under laparoscopy is needed to achieve sufficient mobilization.

Secondly, it is necessary to elaborate on the significance of IA and EA for patients undergoing complex mobilization surgery with adequate freeing. Laparoscopic surgery may inevitably involve flipping and stretching the residual bowel and mesentery, which may lead to damage to partial damage around the intestine in such patients. Theoretically, this may affect the recovery of postoperative intestinal peristalsis and other aspects.

Thirdly, consideration should also be given to nursing factors after anastomosis. Compared to surgical procedures, the impact of postoperative care on efficacy is equally important but often overlooked. Enhanced recovery after rectal and colon surgery has been widely recognized and promoted^5^. Teramura *et al*. described preoperative preparation, intraoperative anesthesia, and other protocols but did not mention postoperative care plans. Suggest adding explanations for postoperative care, such as the timing of early extubation, sham feeding, and early mobilization, to eliminate potential confounders.

This study adopted rigorous methods, including explicit inclusion and exclusion criteria and comprehensive outcome measurements for anastomosis, providing valuable insights for non-invasive laparoscopic surgery. However, the above suggestions can enhance credibility and explanatory power in future research.

## Ethical approval

Not applicable.

## Consent

Not applicable.

## Sources of funding

Science and Technology Plan Project of Taizhou (22ywb07).

## Author contribution

T.H.: investigation, conceptualization, and writing – original draft; S.L.: supervision, funding acquisition, and writing – review and editing.

## Conflicts of interest disclosure

There are no conflicts of interest.

## Research registration unique identifying number (UIN)

Not applicable.

## Guarantor

Shuai Liu.

## Data availability statement

Not applicable.

## Provenance and peer review

Not applicable.
